# Enhancing Antioxidants Extraction from Agro-Industrial By-Products by Enzymatic Treatment

**DOI:** 10.3390/foods11223715

**Published:** 2022-11-18

**Authors:** Paulina Leite, Isabel Belo, José Manuel Salgado

**Affiliations:** 1 CEB—Centre of Biological Engineering, University of Minho, 4710-057 Braga, Portugal; 2 LABBELS—Associate Laboratory, 4710-057 Braga/4804-533 Guimarães, Portugal; 3 Biotecnia Group, Department of Chemical Engineering, Campus Agua, University of Vigo (Campus Ourense), 32004 Ourense, Spain

**Keywords:** lignocellulolytic enzymes, antioxidants, thermostability, circular economy

## Abstract

Nowadays, agro-industrial by-products are of increasing interest as a source of antioxidant compounds. Thus, alternative green techniques to extract antioxidant compounds have been pursued. The use of enzymes to release bioactive compounds through antioxidant activity reduces the environmental impact caused by traditional extraction systems using organic solvents. A crude enzymatic extract containing carbohydrolases was produced by solid-state fermentation (SSF) of an olive pomace and brewery spent-grain combination. The crude extract was evaluated at different temperatures and pH values and its thermostability was studied. Results showed that β-glucosidase and cellulase were more stable than xylanase, particularly cellulase, which kept 91% of its activity for 72 h at 45 °C. The extract was also applied in enzymatic treatments (ET) to liberate antioxidant compounds from winery, olive mill and brewery by-products under optimal conditions for enzymatic activities. The highest antioxidant activity was found in extracts obtained after enzymatic treatment of exhausted olive pomace (EOP). Enzymatic crude extract produced by SSF was successfully applied in the extraction of antioxidant compounds from winery, olive mill and brewery by-products. Thus, integrating SSF and enzymatic technologies is a valuable approach to implement circular economy practices in the agro-food industry.

## 1. Introduction

The wine, beer and olive oil industries face a great challenge to transform their linear economy into a circular economy. Every year, these industries generate large amounts of by-products in short periods of time, which require appropriate management. Allied Market Research predicts that the global antioxidants market is expected to reach USD 4531 million by 2022, registering an annual growth rate (CAGR) of 6.42% [1] (Allied Market Research, 2022). In 2020/21, the global production of olive oil accounted for approximately 3.2 million metric tons [2]. On the other hand, the three largest wine producers (Italy, France and Spain) produced 13.64 Mm^3^ in 2020 [2] and the ten countries leading beer production (China, US, Brazil, Mexico, Germany, Russia, Japan, Vietnam, Poland and Spain) produced 115.76 m^3^ [2].

There are different potential applications for the by-products of the wine, beer and olive oil industries, such as their use in the production of bioproducts with applications in the food, chemical and pharmaceutical industries. Olive mill and winery by-products are a good source of antioxidant compounds [3]. These compounds are of particular significance to human health, since they avoid the increase in reactive oxygen species, which unbalance the “oxidative status”, a critical factor in the pathogenic processes of various chronic disorders [4]. They are aromatic compounds that are divided into several families: acids, phenols, anthocyanins, tannins, lignins, coumarins, flavonoids and quinones [5]. Crude extracts from plants are rich in phenolics and are of increasing interest for the food industry, since they improve the quality and nutritional value of food [6].

Phenolic compounds in plant tissues occur in free, esterified and insoluble-bounded forms [7]. Bounded phenolic compounds that are insoluble have low bioavailability. Therefore, there is a need to select suitable processes to increase the bioavailability of phenolics by facilitating their release, especially the insoluble-binding phenolic fractions [8]. 

Conventional techniques require the use of organic solvents, temperature and agitation, such as the Soxhlet procedure, maceration and hydrodistillation. Recently, other techniques have been proposed, which are greener and cleaner since they require less energy and reduce or substitute the use of organic solvents, making them beneficial to the environment [9,10].

Extraction/production of phenolic compounds by solid-state fermentation (SSF) has also been reported to bean eco-friendly process, because the microorganisms involved modify raw materials, releasing the cross-linked phenolic compounds by the hydrolytic action of several enzymes segregated to the medium during fermentation [11].

Plant cell wall-degrading filamentous fungi produce a broad variety of extracellular enzymes with diverse catalytic activities that are ideal for the hydrolysis of renewable lignocellulose-containing raw materials. The extracellular enzymatic system includes: hydrolytic enzymes, which are responsible for polysaccharide degradation and oxidative enzymes, which degrade lignin and open phenyl rings [12]. Several studies have shown that lignocellulolytic and carbohydrate-hydrolysing enzymes produced during SSF, such as pectinases, α-amylase and α/β-glucosidase, have a positive influence on the mobilisation of soluble phenolics [13,14,15].

The main objective of this work was to extract antioxidant compounds from by-products of the wine, beer and olive oil industries using eco-friendly extraction technologies such as enzymatic treatment (ET). Firstly, an enzymatic extract was produced by *Aspergillus niger* CECT 2088 using SSF of agro-industrial by-products (Brewer’s spent grain and olive pomace). The enzymatic extract was characterised by the effect of temperature and pH on enzymes’ activity and the thermostability of the enzymes. Then, the ET was performed on four untreated agro-industrial by-products to extract antioxidant compounds and reduce sugars. In addition, sequential SSF and ET was also evaluated as a novel strategy to extract antioxidant compounds.

## 2. Materials and Methods

### 2.1. Material

Vine trimming shoots (VTS) of the variety “Treixadura” were obtained from a winery belonging to Origin Designation of Ribeiro (Ourense, Spain). Exhausted olive pomace (EOP) and crude olive pomace (COP) were gathered from an olive mill in Vila Flor and Vila Real, Portugal, respectively. Brewer’s spent grain (BSG) was collected from a brewing plant in Portugal. The humidity of these by-products was reduced at 65 °C in an oven for a day and preserved in dry conditions until further usage.

### 2.2. Microorganisms 

Filamentous fungus used in this experiment was *Aspergillus niger* CECT 2088, acquired from the culture collection of CECT (Colección Española de Cultivos Tipo, Valencia, Spain) and maintained on potato dextrose agar (PDA) plates at 4 °C. The fungus was grown on PDA at 25 °C until a dense sporulation was observed.

### 2.3. Solid-State Fermentation

SSF process was carried out as described by [3]. An optimal mixture of agro-industrial by-products (42% COP, 46% BSG and 12% EOP) was used as the substrate. At the end of each run, enzymes and phenolic compounds of the fermented and unfermented solids were extracted with water (5 mL per g of dry substrate). They were mixed for 30 min without temperature control. Afterwards, the solid and enzymatic extracts were separated by filtration and centrifugation at 4000× *g* for 10 min and 4 °C.

### 2.4. Characterisation of the Enzymatic Extract

The effects of temperature and pH on xylanase, cellulase and β-glucosidase activities were studied. Temperatures between 30 °C and 70 °C, in intervals of 5 °C were evaluated, fixing the pH with 0.1 M sodium acetate buffer (pH 4.6). Different pH ranges from 3.0 to 6.0 (0.1 M sodium acetate buffer) were tested, fixing the optimal temperature determined for each enzyme. The thermostability of enzymes was determined by measuring the residual activity under standard assay conditions after incubation at 40 °C, 45 °C and 50 °C for 72 h.

### 2.5. Enzymatic Treatment (ET)

The enzymatic treatment was performed according to the method described by Fernandes et al. [16] with some modifications. Experiments were carried out in 100 mL Erlenmeyer flasks with 1 g of untreated agro-industrial by-products (COP, EOP, BSG and VTS) and 30 mL of sodium acetate buffer (0.1 mol L^−1^, pH 4.6) was added. The crude enzymatic extract load was 0.75 mL extract/g solid, which corresponded to 150 U of xylanase, 91 U of cellulase and 207 U of β-glucosidase per g of dry substrate. ETs were performed in an orbital shaker at 45 °C for 72 h under constant agitation (150 rpm). At each defined time interval, two samples were taken, centrifuged at 4000× *g* for 10 min at 4 °C and stored at −20 °C. 

Different concentrations of enzymes were tested, using several crude extract loads with 0.75, 1.5, 2.5 and 5.0 mL extract/g solid under the same above-mentioned conditions of temperature, agitation and incubation time. 

### 2.6. Sequential SSF and ET

This treatment was performed according to the method used by Fernandes et al. [17] with some modifications. SSF was carried out according to Section 2.3 but using 5 g of substrate mixture. After 7 days of SSF, 150 mL of sodium acetate buffer (0.1 mol L^−1^, pH 4.6) was added to the fermented mixture and was incubated in an orbital shaker at 45 °C for 72 h under constant agitation (150 rpm). Total phenolic compounds (TPC), antioxidant activity and sugar concentration in supernatants were analysed. 

### 2.7. Analytical Methods

TPC were measured by the Folin–Ciocalteu method (Commission Regulation (EEC) No. 2676/90). The TPC units were defined as mg of gallic acid equivalents (GAE) per g of dry solid (ds). All the analyses were performed in duplicate.

Antioxidant activity was determined following the method described by [18] using DPPH as substrate. The antioxidant activity of the extracts was defined as micromoles of Trolox equivalents per gram of dry solid (μmol TE/g).

The β-glucosidase, cellulase and xylanase activity was measured using the method described by [19]. 

All enzymes’ activities were expressed as units per gram of dry substrate (U/g).

Sugars’ concentration in the liquid phase was quantified using a high-pressure liquid chromatography (HPLC) system using an intelligent refractive-index detector (Jasco830-IR, Jasco, Easton, MD, USA) and a column (Varian MetaCarb 87H, Agilent Technologies Santa Clara, CA, USA). The column was eluted with 0.005 M H_2_SO_4_ and the flux was 0.7 mL/min at 60 °C.

### 2.8. Statistical Analysis

Results are presented as mean ± standard deviation (SD) and were calculated from the data obtained from two independent experiments. Statistically significant differences of means were evaluated with a one-way analysis of variance (ANOVA). A significant difference was considered if *p* < 0.05 using Tukey’s multiple comparisons test. Statistical analyses were performed using Statgraphics Plus 5.1 (Manusgistics, Inc., Rockville, MD, USA).

## 3. Results and Discussion

### 3.1. Enzymatic Characterisation

The enzymatic extracts produced by *A. niger* via SSF of a mixture of by-products from olive mills and breweries were characterised by optimum temperature and pH and thermostability.

#### 3.1.1. Effect of Temperature and pH on Enzyme Activity

**Figure 1:**
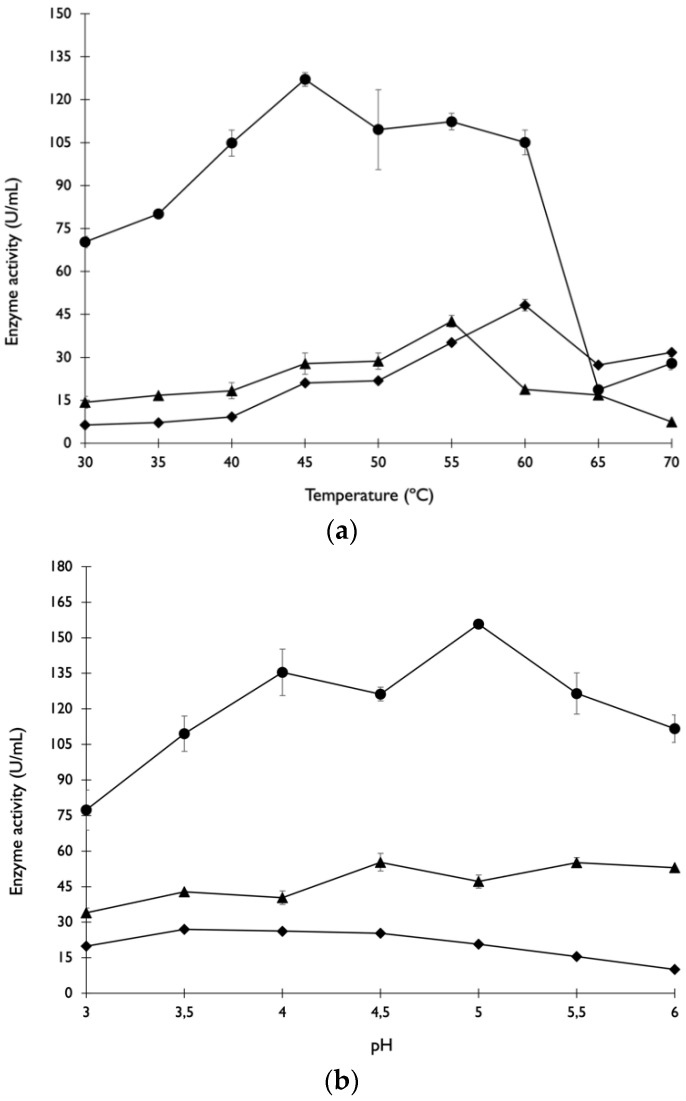
Effects of temperature (**a**) and pH (**b**) on xylanase activity (●), cellulase activity (▲) and β-glucosidase activity (◆). Letters in each value indicate the results of Tukey’s test (*p* < 0.05).

The effect of temperature on the activity of enzymes in the crude extract is presented in Figure 1a. The highest values of xylanase activity were observed in the range of 40 °C to 60 °C, with a significant drop at 65 °C and above. In the case of cellulase and β-glucosidase, the optimal activity was observed at specific temperatures of 55 °C and 60 °C, respectively. β-glucosidase showed low activity in temperatures ranging from 30 to 40 °C, but the decrease in activity at 65 °C was not as pronounced as it was observed for xylanase and cellulase activities. Enzymatic activities from others strains of *A. niger* showed a similar behaviour in the same range of temperatures. Farinas et al. [20] studied the enzymes produced from a selected strain of *A. niger* by SSF of wheat bran, and they observed that the optimum temperature values for endoglucanase, β-glucosidase and xylanase activities were between 35 °C and 60 °C. Xylanase produced by *A. tubigensis* showed maximum activity at the optimal temperature of 50 °C, being reduced at 60 and 70 °C [21]. The crude enzyme cocktail (β-glucosidases, endo-β-1,4-glucanase, exoglucanase and endo-β-1,4-xylanase) produced by SSF of raw oil palm frond leaves using *Rhizopus oryzae* UC2 achieved maximal activities at temperatures between 50 and 60 °C [22]. In other studies, the maximum cellulase activity by *A. niger* BK01 during SSF with alkali-assisted acid-pre-treated rice straw was observed at 40 °C [23], a temperature slightly lower than those reported in the present study. 

The effect of pH on enzymes’ activity is presented in Figure 1b. Maximum xylanase activity was observed at pH values between 4 and 5.5. No statistical differences were observed for cellulase activity at pH values ranging between 3 and 6. The maximum β-glucosidase activity was observed between pH 3.5 and 4.5. Xylanases produced by fungi are genetically single chain glycoproteins, usually with a molecular weight of 15–145 kDa, and are active at an optimal pH ranging between 4 and 6 [24]. According to Elegbede and Lateef [25], the optimum pH to maximise xylanase activity ranged between 5 and 7 during SSF of corn cob. Optimal activities were recorded at pH 6 for xylanases produced by *A. niger* L3. On the other hand, Azzouz et al. [26] reported that the optimum pH for xylanase activity produced from *A. niger* strain BG using wheat bran in SSF was 2.5. Prajapati et al. [27] characterised cellulase from *A. tubingensis* NKBP-55 produced in SSF of copra meal and observed that the optimum activity occurred at pH 5.0. Santos et al. [28] observed that CMCase and β-glucosidase obtained from SSF with *A. niger* 3T5B8 using wheat bran as substrate had optimal activities at pH 3.8 and 4.8, respectively.

Enzymes produced during SSF exhibit optimum activities in wide ranges of temperature and pH; thus, being highly suitable in the saccharification of lignocellulosic materials and/or to extract antioxidant compounds during enzymatic treatments.

#### 3.1.2. Thermostability

**Figure 2:**
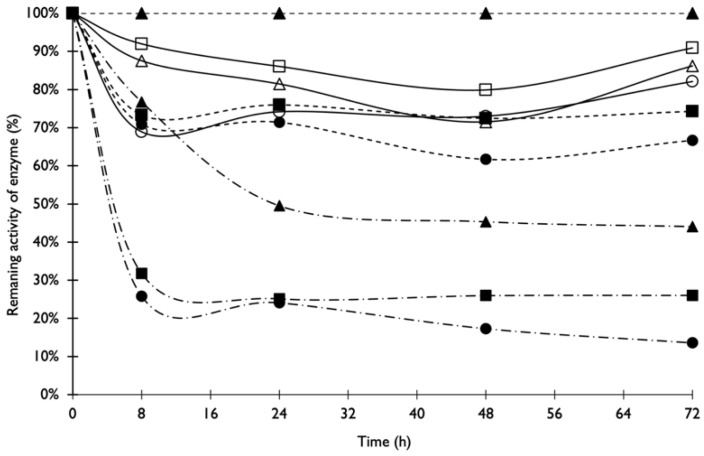
Thermostability of xylanase activity at 40 °C (- . -▲- . -), 45 °C (- . -■- . -) and 50 °C (- . -●- . -); cellulase activity at 40 °C (--▲--), 45 °C (--■--) and 50 °C (--●--); and β-glucosidase activity at 40 °C (△), 45 °C (□) and 50 °C (○).

β-glucosidase produced by *A. niger* was the most resistant to temperature variations, maintaining 82 to 94% of its activity after 72 h at 40 °C, 45 °C and 50 °C (Figure 2). Xylanase lost approximately 50% of its activity after 24 h at 40 °C and more than 70% of its activity after 24 h at 45 °C and 50 °C. Cellulase was stable after 72 h, maintaining 74% and 67% of the initial activity at 45 °C and 50 °C, respectively. On the other hand, cellulase remained continuously stable during the entire incubation at 40 °C.

Ezeilo et al. [22] also evaluated the thermostability of cellulase, xylanase and β-glucosidase (crude enzymatic extract) produced during SSF with *R. oryzae* UC and observed that β-glucosidase produced by SSF in that study was the most thermostable among all assessed enzymes, exhibiting residual activities of 70%, 88% and 64% after 12 h at 40 °C, 50 °C and 60 °C. Kumar et al. [29] characterised purified xylanase from a *Bacillus amyloliquefaciens* strain SK-3; the enzyme showed a considerable activity retention (85%) after 2 h at 50 °C. dos Santos et al. [30] observed that endoglucanase produced by *A. niger* kept its activity during 60 min at 60 °C, decreasing to 20% of its relative activity after 240 min at the same temperature. 

### 3.2. Enzymatic Treatment

**Figure 3:**
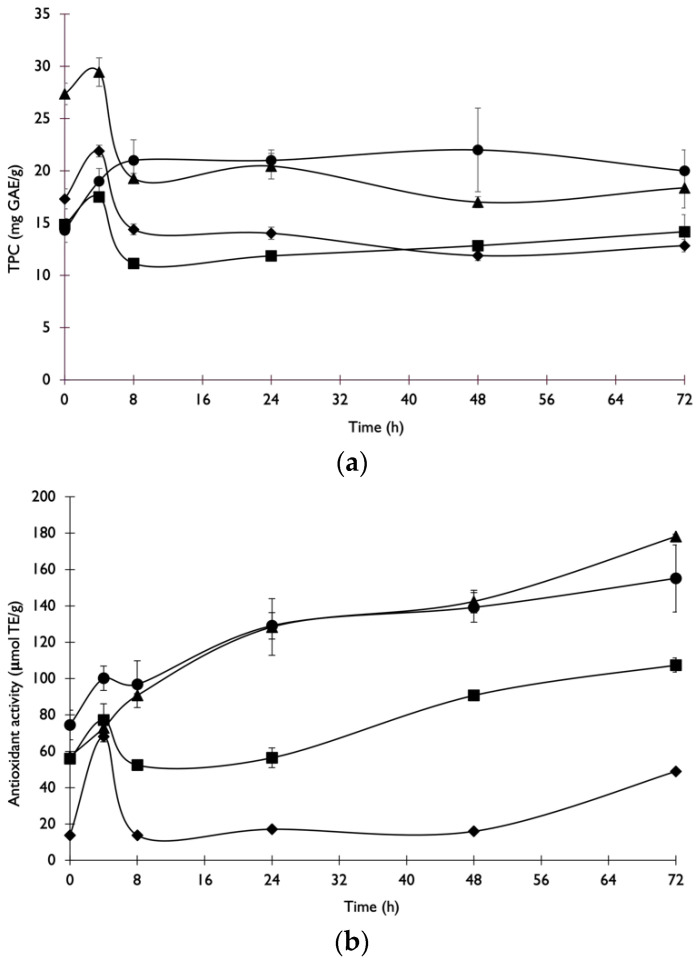
Variation of (**a**) phenolic compounds and (**b**) antioxidant activity after ET of untreated by-products, COP (●), VTS (■), EOP (▲) and BSG (◆), using the enzymatic extract.

The potential of the enzymes produced by *A. niger* during SSF was evaluated with ET of by-products. The crude enzymatic extract was used to enzymatically treat the selected by-products (COP, EOP, BSG and VTS), evaluating the release of TPC, antioxidant activity and sugars (glucose, xylose, and arabinose) during ET. The TPC concentration increased until 4 h and then it decreased (Figure 3a). At the end of the ET, at 72 h, the release of TPC from COP significantly (*p* < 0.05) increased by 38.5%, while a significant reduction in TPC was observed in VTS (25.7%) and EOP (33%) relative to the initial time. No significant differences (*p* < 0.05) were observed in the TPC content of BSG at 0 h and 72 h of ET. The reduction in TPC in VTS and EOP may be related to the degradation of polyphenolics during enzymatic incubation at 40 °C for 48 h [31], since degradation and/or enzymatic polymerisation of the released phenolics by the fermenting fungus may occur during ET [32]. 

All untreated by-products subjected to ET with the SSF enzymatic cocktail increased their antioxidant activity. The highest antioxidant capacity was achieved for the EOP after 72 h of ET (178 ± 1 μM TE/g; Figure 3b). ET increased the antioxidant activity of extracts from all by-products compared to the untreated ones. The highest increase in antioxidant activity was achieved after ET of BSG (3.5-fold), followed by ET of EOP (3.1-fold) and COP (2.1-fold) relative to the initial time. According to Dulf et al. [33], phenolic compounds found in conjugated forms can be released by carbohydrate degrading enzymes, which may also lead to an increase in antioxidant activity. Therefore, the increase in antioxidant activity achieved with ET in this study may be explained by the action of cellulases and xylanases present in the enzymatic cocktail produced by *A. niger,* which promoted the release of phenolic compounds with high antioxidant potential that were previously entrapped in the polysaccharide matrix.

For the four solids used in this study, the amount of glucose and other sugars released by the ET was higher in VTS (65%) and BSG (60%) than in the other by-products. The ET of COP and EOP only released 23% and 37% of total sugars, respectively (Table 1). These by-products are lignocellulosic materials constituted of cellulose, hemicellulose and lignin. ET involves the cleavage of complex polymers of hemicellulose and cellulose using cellulolytic enzymes (cellulases and xylanases). The cellulose is usually only constituted of glucans and, consequently, glucose is the main product released in its hydrolysis. Hemicellulose contains polymers of several sugars, such as xylan, glucan, galactan, arabinan and mannan, and its cleavage yields several pentoses and hexoses. However, high lignin content blocks enzymes accessibility, causing end-product inhibition, and reduces the rate and yield of hydrolysis products [34]. According to Leite et al. [35], BSG has 144 g/kg of lignin, followed by VTS with 341 g/kg, while COP and EOP have 431 g/kg and 550 g/kg, respectively. Therefore, the higher total sugars released in BSG and VTS than in COP and EOP may be due to differences in lignin contents.

#### Crude Extract Load in Enzymatic Treatment

**Figure 4:**
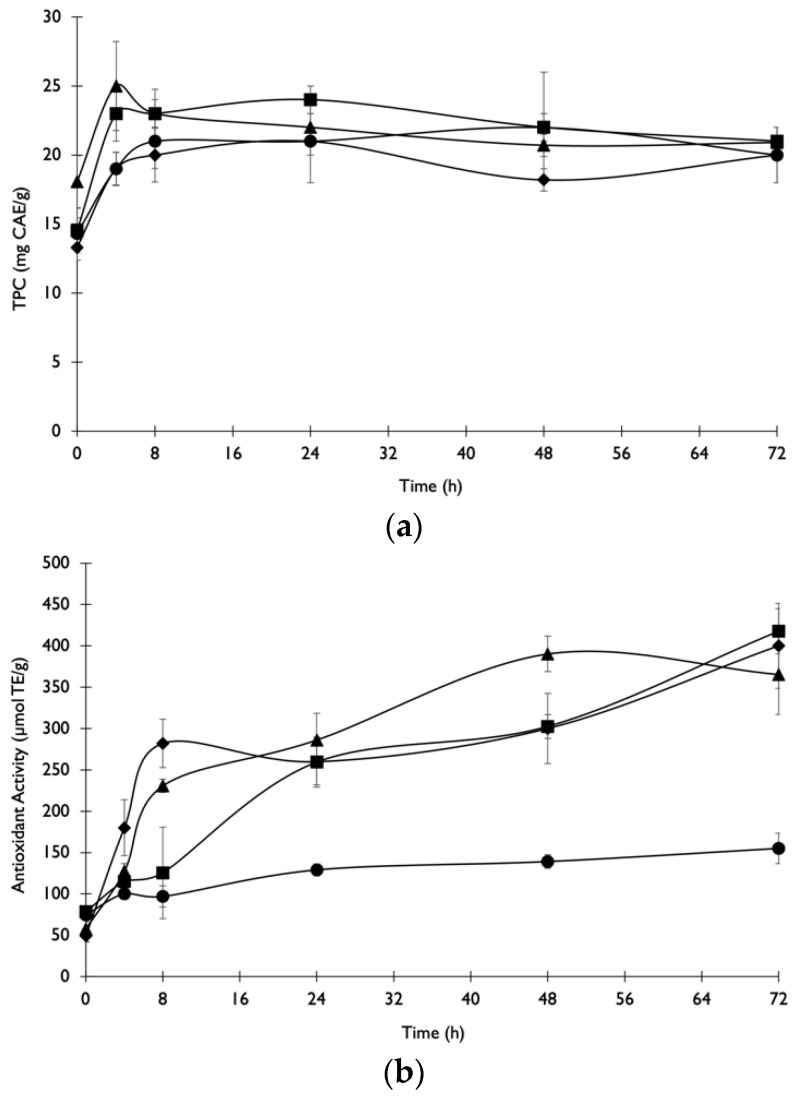
Variation of (**a**) phenolic compounds and (**b**) antioxidant activity after ET of untreated COP using the enzymatic extract with crude extract loads of 0.75 (●), 1.5 (■), 2.5 (◆) and 5 mL extract/g of substrate (▲).

The study of the effect of enzymatic extract load on ET was carried out with untreated COP due to its high potential to release antioxidant compounds. After 8 h, no significant differences (*p* < 0.05) were observed in TPC release between all the experiments with different crude extract loads (Figure 4a). As can be observed in Figure 4b, after 4 h of ET all crude extract loads except for the crude extract load of 0.75 mL/g significantly increased (*p* < 0.05) the antioxidant activity release relative to the initial time. After 8 h of ET, the crude extract loads of 2.5 and 5 g/g (*v*/*w*) improved the extraction of antioxidant compounds compared to the crude extract loads of 0.75 and 1.5 g/g (*v*/*w*) at the same time. After 48 h, the maximum antioxidant activity was achieved using the highest crude extract load. 

Trinh et al. [7] reported that the use of cellulase significantly improved phenolic extraction yields and antioxidant capacities for camellia, rose and roselle as this enzyme was able to hydrolyse the product’s cell walls, facilitating the release and recovery of these compounds. Ghandahari Yazdi et al. [36] also observed an increase in the extraction yield of phenolic compounds along with the increase in the cellulase load. 

Table 2 shows the effect of increasing concentrations of enzymes on the EH of untreated COP. The most suitable enzyme concentration to maximise sugars release (41%) was 2.5 g/g (*v*/*w*). The EH of COP resulted in lower levels of arabinose and higher levels of glucose release. The increase in crude extract load did not increase sugars’ release after 72 h of ET.

### 3.3. Sequential SSF and ET

**Figure 5:**
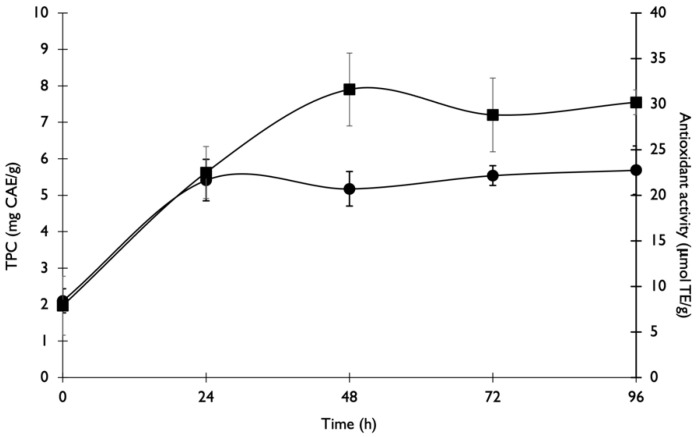
Variation of phenolic compounds (●) and antioxidant activity (■) after sequential SSF and ET.

A sequential SSF and ET were performed on COP to optimise the pre-treatment process by reducing the number of stages required to maximise the release of antioxidant compounds. In this case, the enzymes produced during SSF were not extracted and the fermented COP was immediately submitted to sequential ET with the addition of buffer to the mixture. In Figure 5, time 0 h represents the end of SSF by the addition of buffer, beginning the optimal conditions for ET. The sequential SSF and ET increased the phenolic compounds and the antioxidant activity release from the fermented by-products by 2.7- and 3.8-fold, respectively. The maximum TPC and antioxidant activity release were achieved after 24 h and 48 h of ET, respectively. The maximum antioxidant activity achieved by sequential SSF and ET was lower than that observed by the single ET of untreated COP. The quantity of xylose and arabinose released after 96 h of ET was 3.8 and 11.0 mg/g, respectively, while glucose was not detected. This may be due to its consumption by the fungus. 

## 4. Conclusions

The enzymatic extract produced by *A. niger* during SSF using a mixture of agro-industrial by-products was characterised. The optimum temperature for maximal activity of xylanase ranged between 40 °C and 60 °C. For cellulase and β-glucosidase optimum activities, the specific temperatures were 55 °C and 60 °C, respectively. Maximum xylanase and β-glucosidase activities were observed for pH values between 4 and 5.5 and 3.5 and 4.5, respectively. Cellulase showed maximal activity over a broader pH value range (between 3 and 6), demonstrating that pH variation did not exert such a predominant effect in these enzymes compared to xylanase and β-glucosidase. Cellulase and β-glucosidase produced by *A. niger* were more resistant to temperature variations than xylanase.

The ET of untreated by-products is more effective in releasing phenolic compounds with antioxidant activity than sequential SSF and ET. The highest crude extract load used in the ET was not so effective for obtaining the highest antioxidant activity. ET and SSF are green alternative methods to potentiate the release and utilisation of phenolic antioxidant compounds entrapped in the multiple lignocellulosic fractions of agro-industrial by-products, thereby increasing the bioavailability of these compounds for use in several industrial applications, namely in food and pharmaceuticals, among others. In future works, these extracts with antioxidant activity could be applied as additives in the enrichment of feed or food.

## Figures and Tables

**Table 1:** Sugars released by enzymatic treatment of by-products.

		Glucose (mg/g)	Xylose (mg/g)	Arabinose (mg/g)
COP	0 h	105 ± 7	17.2 ± 0.9	15.2 ± 0.5
	72 h	126 ± 9	21 ± 2	22 ± 1
EOP	0 h	33.80 ± 0.02	21.4 ± 0.2	17.25 ± 0.08
	72 h	49.49 ± 0.03	27.34 ± 0.04	22.7 ± 0.1
VTS	0 h	56 ± 2	39 ± 3	6.3 ± 0.9
	72 h	101 ± 4	55 ± 2	10.307 ± 0.005
BSG	0 h	220 ± 10	23 ± 2	6.81 ± 0.01
	72 h	312 ± 7	69 ± 2	20 ± 1

**Table 2:** Sugars released by enzymatic treatment of untreated COP with different crude extract loads.

		Glucose (mg/g)	Arabinose (mg/g)
0.75 g/g	0 h	103 ± 8	17.1 ± 0.2
	72 h	136 ± 10	24 ± 2
1.5 g/g	0 h	97 ± 9	14 ± 2
	72 h	130 ± 11	22 ± 2
2.5 g/g	0 h	95 ± 9	14 ± 2
	72 h	130 ± 11	22 ± 2
5.5 g/g	0 h	102.2 ± 0.4	21.0 ± 0.8
	72 h	166 ± 17	29 ± 2

## Data Availability

The date are available from the corresponding author.
